# Resuming elective hip and knee arthroplasty after the first phase of the SARS-CoV-2 pandemic: the European Hip Society and European Knee Associates recommendations

**DOI:** 10.1007/s00167-020-06233-9

**Published:** 2020-08-25

**Authors:** N. P. Kort, E. Gómez Barrena, M. Bédard, S. Donell, J.-A. Epinette, B. Gomberg, M. T. Hirschmann, P. Indelli, Ismail Khosravi, T. Karachalios, M. C. Liebensteiner, B. Stuyts, R. Tandogan, B. Violante, L. Zagra, M. Thaler

**Affiliations:** 1 CortoClinics, Schijndel, The Netherlands; 2 Department of Orthopaedic Surgery and Traumatology, Hospital La Paz, Universidad Autónoma de Madrid, Madrid, Spain; 3 Département de Chirurgie Orthopédique, CHU de Québec-Université Laval, Quebec City, QC Canada; 4 Norwich Medical School, University of East Anglia, Norwich, UK; 5 Center for Research and Documentation in Arthroplasty, Lille, France; 6 OA Centers for Orthopaedics, Portland, ME USA; 7 Department of Orthopaedic Surgery and Traumatology, Kantonsspital Baselland, (Bruderholz, Liestal, Laufen), 4101 Bruderholz, Switzerland; 8 University of Basel, Basel, Switzerland; 9 Department of Orthopaedic Surgery, Stanford University School of Medicine, Stanford, CA USA; 10 International Committee American Academy Hip and Knee Surgeons (AAHKS), Rosemont, IL USA; 11 Department of Orthopaedic Surgery, Medical University of Innsbruck, Anichstrasse 35, 6020 Innsbruck, Austria; 12 Orthopaedic Department, University General Hospital of Larissa, School of Health Sciences, Faculty of Medicine, University of Thessaly, Volos, Greece; 13 Department of Orthopedic Surgery and Traumatology, GZA Hospitals, Antwerp, Belgium; 14 Ortoklinik and Cankaya Orthopedics, Ankara, Turkey; 15 Orthopaedic Department, Istituto Clinico Sant’Ambrogio IRCCS Galeazzi, Milan, Italy; 16 Hip Department IRCCS Istituto Ortopedico Galeazzi, Milan, Italy

**Keywords:** COVID-19, Primary joint arthroplasty, Hip, Knee, Safety, Recommendations

## Abstract

**Purpose:**

The Covid-19 pandemic has disrupted health care systems all over the world. Elective surgical procedures have been postponed and/or cancelled. Consensus is, therefore, required related to the factors that need to be in place before elective surgery, including hip and knee replacement surgery, which is restarted. Entirely new pathways and protocols need to be worked out.

**Methods:**

A panel of experts from the European Hip Society and European Knee Association have agreed to a consensus statement on how to reintroduce elective arthroplasty surgery safely. The recommendations are based on the best available evidence and have been validated in a separate survey.

**Results:**

The guidelines are based on five themes: modification and/or reorganisation of hospital wards. Restrictions on orthopaedic wards and in operation suite(s). Additional disinfection of the environment. The role of ultra-clean operation theatres. Personal protective equipment enhancement.

**Conclusion:**

Apart from the following national and local guidance, protocols need to be put in place in the patient pathway for primary arthroplasty to allow for a safe return.

## Introduction

In recent months, the SARS-CoV-2 pandemic has evolved rapidly in Europe, disrupting the personal, social, economic and professional lives of health professionals on a large scale. The overall goal of most governments in Europe has been to flatten the curve of infected SARS-CoV-2 patients and prevent a collapse of national health systems. The April 2020 SARS-CoV-2 survey completed by EHS and EKA members in Europe has confirmed the impact of SARS-CoV-2. This pandemic has resulted in a tremendous reduction in primary hip and knee arthroplasties performed. Consensus is required about the factors that need to be in place before restarting such procedures.

Delaying hip and knee arthroplasty in patients with severe osteoarthritis (OA) may adversely affect their final outcome. It lead to increased opioid use. It is associated with lower clinical results and increased readmission rates after the index procedure. Moreover, when access to hip and knee arthroplasty is limited, as it is now in the wake of the SARS-CoV-2 virus control measures, the direct and indirect costs for health care and social systems are enormous. Many patients suffering from OA have to prolong their absence from work, request temporary unemployment benefits, and/or burden the public welfare system.

A new phase is now starting in most European countries, where restarting elective hip and knee arthroplasty surgery is being planned. The existing guidelines are not robust enough and therefore, improvement is much needed. The aim of this report is to describe the optimal trajectory for restarting elective hip and knee arthroplasty surgery. As a result, the European Hip Society (EHS) and the European Knee Association (EKA) worked with a panel of experts and have produced a thorough consensus report on how safe reintroduction of elective arthroplasty surgery can be achieved. The recommendations are based on the most recent available evidence and have been validated in a survey by world-wide arthroplasty surgeons.

## Method

A panel of experts were tasked with reviewing the evidence available for managing primary elective hip and knee arthroplasty following the SARS-CoV-2 pandemic were asked:

### Five basic questions

1. When should elective hip and knee arthroplasty be resumed?
2. Are new triage/patient selection criteria needed for hip and knee arthroplasty patients once elective surgery is resumes?
3. What are the priority indications for elective adult hip and knee reconstruction?
4. What is the role of outpatient hip and knee arthroplasty/enhanced recovery protocols in light of the SARS-CoV-2 pandemic?
5. What is the impact of delaying hip and knee arthroplasty for the patients themselves and for society?

### Preoperative phase

1. What is the appropriate preoperative clinical and laboratory screening and timeline for patients?
2. Is preoperative tracking of patients, staff and relatives necessary?
3. Should spinal anaesthesia be routine?
  Should only patients who also consent to regional/spinal anaesthesia be selected as candidates?
  If spinal anaesthesia does not occur, should the operation be cancelled?

4. If the SARS-CoV-2 tests are positive, how long should the hip or knee arthroplasty surgery be postponed?
5. What kind of back-up plan is needed to guarantee quality care and patient safety in this pandemic situation?

### Perioperative phase

1. Is there a need for:
  Modification or reorganization of hospital wards (patient density, bed density, medical and nursing stuff density, etc.)?
  Separation of elective and trauma orthopaedic surgery (with regard to the general orthopaedic departments)?
2. Should relatives/visitors or other supportive personnel be allowed onto the orthopaedic wards and operating suite?
3. Is there a need for extra disinfecting environmental procedures in between operations?
4. What roles do ultra-clean operating theatres, orthopaedic exhaust suits and ultraviolet light systems play in both the wards/beds and the theatre?
5. What are the guidelines for personal protective equipment (PPE), in terms of availability and instructions regarding its use and what to wear in relation to the patient’s position in the perioperative chain?

### Post-operative phase

1. Since the impact on mind and body during joint arthroplasty is significant, do the patients may have lower immunity and be more vulnerable to SARS-CoV-2 infection?
2. Should a shelter-in-place post-operative period, home care, community nurses or informal care be organised?
3. Should face-to-face contact for the orthopaedic follow-up and physical therapy guidance be avoided?
  Consider telemedicine
  Consider a new approach in wound closure technique in the setting of the SARS-CoV-2 pandemic
4. What is the advice for patients who develop SARS-CoV-2 symptoms?
5. Are special adjustments needed for post-op SARS-CoV-2 prophylaxis?

The panel then agreed a statement that answered each question, along with reporting the rationale for the recommendation.

## Results

### Five basic questions

1. When should elective hip and knee arthroplasty be resumed?

*Statement* Elective hip and knee arthroplasty can be resumed when appropriate prerequisites concerning facilities, workforce, testing, supplies are met, and approval of local health authorities is obtained. Facilities in areas with low, or relatively low and stable incidence of SARS-CoV-2, can be allowed to provide care for patients needing non-emergency, non-SARS-CoV-2 healthcare.

*Rationale* The US Centers for Disease Control advise that the following gating criteria to be met before reopening facilities to provide non-emergency health care [43]:

- Downward trajectory of cases with influenza-like and COVID symptoms within the last 14 days.
- Downward trajectory of documented cases and positive tests as a percentage of total tests within 14 days
- Hospitals have treated all COVID cases without crisis care
- Robust testing programme in place for at-risk health care workers, including emerging antibody testing

Depending on the country and the locations involved, some hospitals may be overburdened, while non-COVID care facilities may be inactive. The US Centers for Medicare & Medicaid Services (CMS), in their ‘Recommendations for Re-opening Facilities to Provide Non-emergent Non-SARS-CoV-2 Health Care’ [6], advise careful consideration of the availability of adequate facilities, testing and supplies, plus an adequate workforce, across all the phases of care (e.g. clinicians, nurses, anaesthesia, pharmacy, imaging, pathology support and post-acute care).

The International Consensus Group (ICM) and the AAHKS Research Committee advise that the following prerequisites be met before the resumption of elective procedures [29]:

- Timing of resumption: There must be a sustained reduction in the rate of new SARS-CoV-2 cases in the relevant geographic area for at least 14 days before the resumption of elective surgical procedures [6, 29, 32, 43]
- Any resumption should be authorised by the appropriate municipal, county and state health authorities
- Facilities in the state are safely able to treat all patients requiring hospitalisation without resorting to crisis standards of care
- The facility needs to have an appropriate number of ICU and non-ICU beds, PPE items, ventilators, medications, anaesthetics and medical surgical supplies to cope with a second wave of pandemic
- Appropriate decontamination of the hospital and necessary equipment has occurred
- The facility needs to have an adequate number of trained and educated staff available to handle the planned surgical procedures, patient population and facility resources. Given the known evidence supporting health care worker fatigue and the impact of stress, the facilities should be able to perform planned procedures to organise two different pathways inside the structure for SARS-CoV-2 and non-COVID patients without compromising patient safety or staff well-being
- Ideally, all the staff who will be involved in patient care have been tested for SARS-CoV-2 (RT-PCR) on a regular basis and/or undergone serum antibody testing for the virus
- The facility should be able and to have two different, separated surgical blocks for the two categories of patients

ESSKA Guidelines also focus on the SARS-CoV-2 exposure status of the patient described by Fineberg and recommend elective surgery only in the first three of the following six categories [12, 25]:

- Exposure-to-infection status unknown. Should get a COVID RT-PCR test 48–72 h before surgery (plus other means according to your institutional rules and regulations)
- Exposed but asymptomatic. Should get a COVID RT-PCR test, immune/serology test (if allowed and available) and eventually a lung CT scan before surgery
- Recovered from infection and possibly adequately immune. Should get an immune/serology test (if allowed and available) before surgery
- Presumed to be infected (persons with signs and symptoms consistent with infection who initially tested negative). Should get a repeated COVID RT-PCR test, an immune/serology test (if allowed and available) and eventually a lung CT scan before considering any surgeries
- Infected (COVID-kit test—eventually a lung CT scan). Delay any elective surgery for 6 weeks and then should get an immune/serology test (if allowed and available) before surgery
- Infected with co-morbidity (COVID-kit test—eventually a lung CT scan). Delay any elective surgery until full recovery (for at least 2 months) and then should get an immune/serology test (if allowed and available)

2. Are new triage/patient selection criteria needed for hip and knee arthroplasty patients once elective surgery is resumes?
  *Statement* Increased demand for hip and knee arthroplasty, coupled with limited hospital resources, will force surgeons to select which patients should receive hip and knee arthroplasties sooner than others. This will entail employing objective, transparent criteria in prioritising patient selection to identify the patients most in need who also have lower risk factors for disease transmission and post-operative complications.
  *Rationale* An increased demand for hip and knee arthroplasty care is foreseen once elective procedures are resumed in Europe, where public health systems are the major health care payers. Conversely, in countries where health insurance is linked to employment and private insurance companies are the main payers (e.g. the USA), demand may decline due to unemployment/underemployment or people’s need to continue working for economic reasons despite their symptoms. Several guidelines are available for prioritising patients undergoing elective surgery.

The MeNTS Score (Medically Needed Time Sensitive Procedures Score) [32] is a scoring system that takes the following into account:

- Procedural factors (overall procedure time, blood loss, need for ICU, intubation probability)
- Disease factors (viability of non-operative treatment, increased surgical difficulty and risk with delaying the procedure)
- Patient factors (age, cardiopulmonary disease, diabetes, influenza-like symptoms and exposure to COVID-19-positive person)

The score can range from 21 to 105, with higher scores being associated with poorer perioperative patient outcomes, increased risk of SARS-CoV-2 transmission to the health care team, and/or increased hospital resource use. Although there is no threshold for safe elective surgery, the hospitals can adjust their thresholds depending on resources.

The International Consensus Group (ICM) and the AAHKS Research Committee [32] recommend delaying elective surgery for patients over 75 years old with cardiopulmonary co-morbidities, patients with morbid obesity, transplant patients undergoing immunosuppression and patients with active cancer.

ESSKA guidelines [10] advise giving priority treatment to younger patients (< 60), requiring fewer than 3 days of hospitalisation and delaying elective surgery for patients with co-morbidities.

3. What are the priority indications for elective adult hip and knee reconstruction?
  *Statement* There is universal agreement regarding the indications for urgent hip and knee arthroplasty procedures, such as femoral neck fractures, periprosthetic fractures, dislocations and acute infection, even in the setting of the SARS-CoV-2 pandemic. However, priority indications for non-emergency procedures in primary and revision hip and knee arthroplasty are lacking and should be clarified.

EHS and EKA priority indications for elective adult hip and knee arthroplasty:

- Acute fractures requiring hip and knee arthroplasty (periprosthetic fractures, THA/hemi-arthroplasty for femoral neck fractures)
- First-stage explantations for acute PJI (periprosthetic joint infection)
- Amputation in cases of failed arthroplasty
- One-stage revision for acute PJI
- Revision surgery required for massively failed arthroplasty: imminent fracture, massive osteolysis at risk of implant migration, implant breakage, pending fractures because of osteolysis or instability, severe adverse reaction to metal debris (ALTR or ARMD)
- Osteonecrosis with joint collapse, avascular head necrosis (AVN)
- Patient with severe articular deformity and/or instability
- First-stage explantations for chronic PJI
- One-stage revision for chronic PJI
- Patient with severe arthritic condition and/or pain forced to stop working (rapid progressive osteoarthritis)
- Total joint arthroplasty reconstruction after bone sarcoma resection
- Second stage for treatment of an infected joint arthroplasty
- Conversion from osteosynthesis to total joint arthroplasty
- Operations required because prolonged delay could prevent the appropriate surgery from being performed adequately
- Patient with severe functional impairment compromising autonomy and ability to walk
- Patient with inability to walk and requiring important technical aid (wheelchair, home care, human assistance)
- Patient with chronic severe arthritic pain requiring opioid use

*Rationale* Several guidelines outline the urgent indications for hip and knee arthroplasty surgery. The American College of Surgeons’ ‘COVID 19: Elective Case Triage Guidelines for Surgical Care Guidelines on Surgery’ describe the following conditions as priority indications for hip and knee arthroplasty surgery: hip dislocation, knee dislocation, periprosthetic fracture, acute pain exacerbation in prior joint arthroplasty, inability to bear weight on the extremity, wound drainage, fever and concern for periprosthetic infection.

The International Consensus Group and the AAHKS Research Committee recommend priority surgery for impending fracture and exposed implants, in addition to the conditions outlined above.

It would be helpful to have guidelines covering the priority indications for elective surgery for primary hip and knee arthroplasty, with a stratification of patient symptoms, disability, disease severity and soft/tissue bone compromise. Examples might include femoral head collapse in avascular necrosis, severe disability, and painful bone marrow oedema. The same should be done for revision indications, such as bone loss, impending fracture, extensor mechanism disruption, patella dislocation, severe osteolysis, massively failed implants, and second stage implantation of infections.

4. What is the role of outpatient hip and knee arthroplasty/enhanced recovery protocols in light of the SARS-CoV-2 pandemic?

*Statement* It is universally accepted that shorter hospital stays and treatment in a SARS-CoV-2-free environment decrease the risk of SARS-CoV-2 infection in patients undergoing elective surgery. Performing hip and knee arthroplasty with enhanced recovery protocols that decrease the length of stay in a facility or can be used in ambulatory care centres to allow for day surgery might be beneficial for reducing the risk of viral transmission. Transitioning to enhanced recovery systems requires full support, adoption of all the subspecialties involved and the possible approval of health care payers, which may not be possible in the pandemic setting.

*Rationale* Enhanced recovery protocols for hip and knee arthroplasty have been shown to decrease length of stay without increasing complications in appropriate patients [40, 44]. These protocols require a graduated, multi-specialty approach, with refinements being performed according to each centre’s needs [42]. Transitioning to enhanced recovery protocols might be difficult in the setting of the SARS-CoV-2 pandemic, but it must be an intermediate-term goal, involving the health care payers if necessary.

Although the quality of evidence is not very high, outpatient (day case) hip and knee arthroplasties have been shown to be safe and effective in select patients [31]. Medicare has allowed total knee arthroplasties (since 2018) and hip arthroplasties (since 2020) in ambulatory care centres in the USA. With the hospitals burdened with SARS-CoV-2 care, these ambulatory centres can provide the services needed for hip and knee arthroplasty without draining hospital resources. Protocols must be developed for performing hip and knee arthroplasties safely in such centres [24].

The American College of Surgeons’ advice is to adhere to standardised care protocols as much as possible (e.g. enhanced recovery protocols) for increased reliability in light of potential variability in available personnel, since standardized protocols optimise patient safety, minimise hospital stays and increase efficiency. They are associated with decreased complication rates.

5. What is the impact of delaying hip and knee arthroplasty for the patients themselves and for society?

*Statement* Delaying elective hip and knee arthroplasty has negative consequences on the quality of life of patients and negatively impacts society as a whole. The benefits of hip and knee arthroplasty should be carefully weighed against the risks of viral transmission and infection, complications and mortality in the mostly elderly population requiring joint arthroplasty.

*Rationale* In the face of the SARS-CoV-2 pandemic, elective hip and knee arthroplasty procedures may have been delayed or cancelled by the health authorities or by patients fearing viral spread. The consequences of delayed hip and knee arthroplasty for patients have not been studied extensively. A survey of 360 patients with cancelled arthroplasties by Brown et al. [5] showed that daily symptoms of arthritis were unchanged or exacerbated in most patients and limitations at home were significant for 15–20% of them. This study also found that not knowing when the procedure would be rescheduled caused moderate to severe anxiety in 60% of patients. Similar findings were documented by D’Apolito [9]. Becoming infected with SARS-CoV-2, and spreading the infection to others, were also high causes of anxiety. Regardless of the SARS-CoV-2 pandemic, delaying hip and knee arthroplasty is also associated with increased disability, deterioration of function, less benefit from surgery and worse functional recovery in the early post-operative period [14, 34].

The SARS-CoV-2 pandemic has placed an enormous economic burden on the global economy. The effect of any economic downturn is usually a decrease in elective procedures [13]. This decrease is exacerbated in the present situation by fears of viral spread, limitations of travel and stay-at-home precautions enforced at different levels in various countries. The direct effect of delaying hip and knee arthroplasty in the face of the SARS-CoV-2 pandemic is not known. Previous work has shown that both hip and knee arthroplasty have been shown to have societal benefits in terms of increased productivity and earnings and decreased disability pay and missed workdays [18, 35]. A Markov analysis of total hip arthroplasty in Germany by Mujica-Mota et al. [26] showed that the expected benefit of having timely THR is substantial for patients in terms of health related to quality of life, ranging from the equivalent of an additional 3.15 years of life in full health for a 65-year-old male person to 3.93 extra years for a 55-year-old woman. These benefits may be lost if elective procedures are delayed for longer periods of time.

### Preoperative phase

1. What is the appropriate preoperative clinical and laboratory screening and timeline for patients?

*Statement* All patients undergoing surgery should have their temperature and oxygen saturation measured [2]. A thorough medical history should be acquired and patients asked about any symptoms they may have, such as cough, fever, loss of smell or taste, headache or gastrointestinal disorders. Additionally, they should be questioned about recent travels and their occupation to stratify them into possible high-risk groups. Regarding the laboratory testing, it would be ideal for all patients to undergo RT-PCR testing for SARS-CoV-2 before the operation [30]. However, local guidelines and the efficacy of tests must also be taken into consideration. If there is a paucity of available tests, then only high-risk patients should be tested. There is no indication for additional chest CT scans in the preoperative screening. Time allowing, all surgical patients should apply social distancing principles for two full weeks prior to testing and the surgical procedure and self-quarantine in their home for the period between test acquisition and day of surgery.

*Rationale* It is known that patients who remain asymptomatic (30% of exposed cases) or mildly symptomatic (56% of exposed cases) can transmit the infection. Taking these facts into consideration, patients who are scheduled for surgery should always be assumed to be potential carriers of the virus throughout the duration of their hospital or clinical stay, even if they pass the pre-assessment triage, including normal temperature, no history of exposure or travel and no respiratory symptoms [2]. Not being aggressive with testing while carrying out surgical services could have catastrophic consequences.

There are two categories of SARS-CoV-2 tests: tests that detect the virus itself (viral ribonucleic acid [RNA]) and tests that detect the host’s response to the virus (serological antibodies) [30]:

- Nucleic acid amplification tests for viral RNA: Detection of the virus is achieved by identifying the viral RNA through nucleic acid amplification, usually using a polymerase chain reaction (PCR). The most commonly tested sample types are swabs taken from the nasopharynx (more sensitive) and/or oropharynx. Swabs are then placed into a liquid to release viral RNA into the solution and subsequently amplified using reverse transcription PCR. These tests are the gold standard for acute illness. Accuracy of the test is affected by the quality of the sample, and RNA may degrade over time. Since SARS-CoV-2 can infect anyone and result in transmission prior to the onset of symptoms, or possibly even without individuals ever developing symptoms, aggressive testing/screening of asymptomatic patients has been considered.
- Antibody detection via serology: The other broad category of tests include those detecting IgM, IgA, IgG or total antibodies. Clinicians should note that SARS-CoV-2 is not typically present in blood. An infection causes white blood cells to make antibody proteins that help the immune system identify the virus and stop it or mark infected cells for destruction. Development of an antibody response to infection is host-dependent and takes time. In the case of SARS-CoV-2, studies suggest that most of the patients seroconvert in 7–11 days after exposure to the virus, although some patients may develop antibodies sooner. Therefore, antibody testing is not useful in the setting of an acute illness. It is still unknown whether individuals infected with SARS-CoV-2 who subsequently recover will be protected, either fully or partially, from future infection with SARS-CoV-2 or for how long the protective immunity may last.

The Dutch Medical Federation states in their guidelines that patients reporting no symptoms of SARS-CoV-2 after a thorough history taking may be either without infection, asymptomatic or pre-symptomatic. An asymptomatic patient carries the SARS-CoV2 virus but never experiences noticeable symptoms. A pre-symptomatic patient experiences no symptoms of SARS-CoV-2 at the time of evaluation but will go on to subsequently develop SARS-CoV-2 symptoms in the future. When a patient reports no symptoms of SARS-CoV-2 during preoperative screening, it is therefore impossible to make a clinically relevant distinction between the three categories of no SARS-CoV-2 infection, asymptomatic SARS-CoV-2 infection, or pre-symptomatic SARS-CoV-2 infection. This means that patients who are initially asymptomatic, pre-symptomatic or mildly symptomatic can subsequently develop moderate to severe SARS-CoV-2 disease, placing them at significant risk for adverse post-operative outcomes, that is, ICU admittance or increased mortality [3, 22].

Concerning the chest CT, the Dutch Medical Federation’s recent advice is outlined as follows. The committee’s primary aim for the work-up for SARS-CoV-2 is to identify SARS-CoV-2-positive cases (high sensitivity) to limit transmission by rescheduling surgery, if possible, or allowing necessary precautions to be taken. Therefore, the committee initially advised that all adult patients requiring a surgical procedure under general anaesthesia undergo preoperative screening for SARS-CoV-2 infection through the use of SARS-CoV-2 PCR of a deep nasopharyngeal swab, in conjunction with a low-dose chest CT (without i.v. contrast). Preliminary data show 1–2% SARS-CoV-2 infection among preoperative patients reporting no symptoms of SARS-CoV-2. This may seem a low percentage. However operative health care workers undergo repeated exposure during aerosol-generating swab. However, the added value of chest CT is low. Therefore, the committee is of the opinion that there is no longer an indication procedures and other risk-bearing procedures. The cumulative exposure to SARS-CoV-2-positive patients for these health care workers through aerosolisation is therefore assumed to be high. In addition, transmission to other patients remains an unknown risk factor. The committee’s opinion is that this percentage justifies preoperative testing through the use of SARS-CoV-2 PCR of a deep nasopharyngeal for the use of chest CT in preoperative testing of asymptomatic patients.

ESSKA guidelines focus on the SARS-CoV-2 exposure statuses of patients described by Fineberg. Risk factors (e.g. age > 60 years, obesity, high blood pressure, cardiovascular disease and diabetes) are disqualifying conditions in this early phase (and should always be discussed with the anaesthetist). The ESSKA is recommending a preoperative questionnaire; detailed discussions with the patient about their situation (e.g. by tele- or videoconference); and a SARS-CoV-2 RT-PCR test and/or immune/serology test 48 to 72 h before the operation.

OrthoEvidence recommends the screening and detection of SARS-CoV-2 patients for the safe introduction of elective surgery and preservation of vital supplies.

The International Consensus Group (ICM) and the AAHKS Research Committee recommend that testing of patients should be mandatory in high-prevalence areas, given the risk of disease transmission by asymptomatic patients. Routine testing is not feasible in all locations because of limitations in testing capacity, though, and therefore local guidelines should be followed in those areas.

2. Is preoperative tracking of patients, staff and relatives necessary?
  *Statement* All patients and staff will need to be screened for potential symptoms of SARS-CoV-2 prior to entering a hospital facility. In particular, staff must be routinely screened for potential symptoms. Isolation prior to surgery can be guided. All patients, staff and relatives, especially those with patient contact, need to be investigated for previous symptoms, travels abroad and possible contacts with populations at high-risk for SARS-CoV-2.

*Rationale* Park et al. [28] describe an outbreak at a call centre in South Korea. Out of 1143 people, 97 were COVID-19 positive, 94 of whom were from the building. The proportion of pre-symptomatic persons was 4%, and none of the housemates of these persons became infected (versus 16.5% of the housemates of symptomatic persons). The authors concluded that easily accessible testing of symptomatic persons, contact tracing and taking isolation measures were the preferred methods of control. Effective screening of patients and caregivers is necessary to prevent post-operative development of severe COVID-19 disease in a vulnerable patient population.

The ESSKA guidelines and the International Consensus Group (ICM) and AAHKS Research Committee recommendations have a preoperative questionnaire included which can be used for COVID-19 screening.

3. Should spinal anaesthesia be routine?
  *Statement* Spinal anaesthesia can be considered safer for every negatively screened patient (with or without a SARS-CoV-2 test) than general anaesthesia, since the latter requires airway manipulation and endotracheal intubation, procedures that can more easily transmit SARS-CoV-2 [23].

- Should only patients who also consent to regional/spinal anaesthesia be selected as candidates?
  *Statement* It cannot be made mandatory that patients consent to regional/spinal anaesthesia. Nevertheless, the benefits of regional anaesthesia should be thoroughly explained to the patients, and whenever possible, this should be strongly considered as the preferred means of anaesthesia.

*Rationale *General anaesthesia, which requires airway manipulation, endotracheal intubation and positive ventilation, is more predisposed to transmitting SARS-CoV-2 to the anaesthetic and surgical teams. Transmission can occur from asymptomatic patients.

- If spinal anaesthesia does not occur, should the operation be cancelled?

*Statement* If general anaesthesia cannot be avoided, every precaution should be taken to avoid contamination. If a SARS-CoV-2-negative environment has been achieved that is as secure as possible, then general anaesthesia with informed consent can be considered.

*Rationale* Cancellation of surgery is a constant, agonising dilemma for nearly all health care services in terms of the wasting of resources and inconvenience caused to both patients and families. Deciding to cancel a hip or knee arthroplasty surgery means rescheduling long-anticipated, life-changing surgery for the patient. Patients undergoing elective surgery should be educated as to the spinal/general anaesthesia protocols that are in place for minimizing SARS-CoV-2 transmission to themselves, their family members, other patients and hospital personnel.

OrthoEvidence clearly states that we need further research on identifying the ideal screening process for asymptomatic surgical patients, so in general anaesthesia cases, we should consider taking additional measures beyond those required for an operation under spinal anaesthesia.

The American Society of Anesthesiologists’ guidelines suggest that the anaesthetic professional should consider measures that limit the potential for aerosolisation of droplet particles. The following should be considered for all patients:

- Surface and equipment cleaning during and between cases (e.g. have a rigid protocol for anaesthetic machine interface, bag, monitors, surfaces, door handles, etc.; avoid unnecessary clutter)
- Wear gloves (change regularly and when soiled)
- Regular hand washing and avoid contamination of mucus membranes (gloved hands may remind you to not touch your mucus membranes)
- Avoid high-flow devices, especially if not wearing PPE
- Consider Video Laryngoscopy for intubation to distance yourself from the airway and/or wear mask and eye protection, sheath all reusable equipment where possible and ensure appropriate disinfection procedures

The International Consensus Group (ICM) and the AAHKS Research Committee recommend the use of regional anaesthesia whenever possible and say it should be strongly considered for patients undergoing elective surgery during the pandemic. For patients undergoing elective surgery, education is mandatory on the protocols that are in place to minimise SARS-CoV-2 transmission to themselves, their family members, other patients and hospital personnel. An overview of the protocols implemented by the hospital to reduce the risk of transmission of the infection should be included.

The advice of the American College of Surgeons is to consider the guidelines in place, for personnel to be present during intubation, and to consider including a waiting period before beginning the operation to account for air circulation cycling time.

4. If the SARS-CoV-2 tests are positive, how long should the hip or knee arthroplasty surgery be postponed?

*Statement* It is reasonable to assume that patients who test positive for SARS-CoV-2 should not be operated on and should be discharged from the hospital. These patients should remain in quarantine at least 14 days and until a subsequent test turns out negative (two confirmed negative PCR swab tests with a 24 h interval) and they are free of fever, cough or other symptoms.

*Rationale* Obviously, if patients show a positive result for the PCR test, they should be isolated, the national SARS-CoV-2 public health protocols should be followed (including protocols for exposed staff) and the operation should be postponed. There is no exact advice at this point with regard to the length of the postponement. Noting that hip and knee arthroplasty is elective surgery and that the patient’s safety should be optimised, then 3 weeks should be adequate, provided they do not become symptomatic.

The American College of Surgeons’ advice is to have facility-specific SARS-CoV-2 testing policies which would then account for how a facility will respond to a SARS-CoV-2-positive worker, SARS-CoV-2-positive patient (identified preoperative or identified post-operative), a ‘person under investigation’ (PUI) worker or a PUI patient.

The CDC protocol is that if an asymptomatic patient tests positive for SARS-CoV-2, their operation should be rescheduled/cancelled until at least 10 days after their first positive SARS-CoV-2 test, assuming they do not subsequently develop symptoms. If they have symptoms, their operation should be rescheduled or cancelled until it has been at least 3 days since the resolution of their fever without fever-reducing medications and 3 days since any respiratory symptoms (cough, shortness of breath, etc.) have improved and they either:

- Test negative from at least two consecutive NP swab specimens collected > 24 h apart or
- At least 7 days have passed since symptoms first appeared.

5. What kind of back-up plan is needed to guarantee quality care and patient safety in this pandemic situation?

*Statement* The treatment of SARS-CoV-2 patients is placing a huge strain on the resources of many hospitals, to the detriment of treatment of other health problems. Safety of hip and knee arthroplasty patients remains of paramount importance. Complications of cardiac and respiratory origin are the most common problems needing acute care. As elective operations begin, every hospital should have ICU capacity available to accept and accommodate patients with cardiopulmonary or other significant complications. It should be assumed that, after the resumption of normal functions at hospitals and in society in general, there will still be a risk of another outbreak of the disease. In such a situation, hospitals should be prepared to return to a “safe mode” with reorganisation of the wards and personnel. Every hospital should have in place a plan for emergency distribution of the personnel and wards before elective surgery resumes.

*Rationale* Co-morbidities that are common in surgical populations, including hypertension, cardiovascular disease, COPD, asthma and malignancies, also place patients at significantly higher risk of severe SARS-CoV-2 disease [15].

The advice of the American College of Surgeons is to ensure that a post- SARS-CoV-2 outbreak, elective surgery surge will not overwhelm local facilities throughout the preoperative, intra-operative, post-operative and post-acute care phases.

### Perioperative phase

1. Is there a need for:
  Modification or reorganisation of hospital wards (patient density, bed density, medical and nursing stuff density, etc.)?

*Statement* In most orthopaedic departments, the workforces have been adjusted to accommodate fewer cases, due to the reduction in trauma cases and cancellation of elective procedures. As elective operations restart, the standard social distancing guidelines should be sufficient. Patient numbers in the clinical areas (wards, waiting areas, outpatient clinics) should be halved, with the patients spaced at least 2 m apart.

*Rationale* The International Consensus Group (ICM) and the AAHKS Research Committee recommend the following (bearing in mind that not every patient may have had a SARS-CoV-2 test):

- Patients should be housed in single rooms, if possible. When patients are housed in the same room, the beds should be spaced at least 2 m (6 ft) apart, and all patients should wear a surgical mask
- Commonly touched surfaces should be wiped down and cleaned with an effective disinfectant solution (e.g. 70% alcohol) at least twice a day
- Any close contact with the patient should be done with the use of appropriate PPE, as directed by the institutional policy and in conjunction with national and local guidelines
- In the early weeks following the return to elective surgery, each patient should be placed in a single room or cubicle where check-in, registration and other administrative tasks are performed.
- Consideration should be given to having a screening ward to house patients, especially those who may not have been tested for SARS-CoV-2 preoperatively
- Large surgical bays with multiple patients sharing one area should be avoided

- Separation of elective and trauma orthopaedic surgery (with regard to the general orthopaedic departments)?

*Statement* SARS-CoV-2 screening practices should ensure the health and safety of patients and staff. SARS-CoV-2-negative elective hip and knee arthroplasty must be separated from the SARS-COV-2-positive trauma unit. Since all patients undergoing elective procedures are screened before administration, and have tested negative for SARS-COV-2, they may be reluctant to go into certain settings for fear of viral transmission. There should be a physical separation of SARS-COV-2-positive and SARS-COV-2-negative patients and specific wards should be exclusively dedicated to treating patients with SARS-CoV-2 to reduce the risk of contaminating other patients.

*Rationale* OrthoEvidence recommends separating, when possible, known or suspected SARS-CoV-2 patients from other patients (i.e. designating either SARS-CoV-2 institutions or specific clinical areas, wards and operating theatres).

2. Should relatives/visitors or other supportive personnel be allowed onto the orthopaedic wards and operating suite?

*Statement* Until normality returns, the number of visitors should be minimised as much as possible to reduce potential transmission of SARS-CoV-2 among patients, their family members and medical staff. This includes sales representatives who travel to facilities to undergo, perform and support procedures. Limiting interactions between individuals and social distancing are part of the mainstream management against the spread of SARS-CoV-2. The number of visitors should be limited to those who are essential for a patient’s care. Although it is recognised that these rules create considerable anxiety for the patient, keeping all patients, relatives and staff safe from SARS-CoV-2 is the priority.

*Rationale* All these people, including the medical device and implant manufacturer representatives, can transmit the SARS-CoV-2 virus not only from person to person at the patient’s hospital, but to other health care facilities they may visit.

The International Consensus Group (ICM) and the AAHKS Research Committee recommend that:

- The use of waiting rooms should be minimised. Social distancing in the waiting rooms and other communal areas should be exercised. Frequent cleaning is also recommended
- Family members and visitors should limit the time they spend in the hospital. Some institutions may ban the entry of family members and visitors into the hospital
- The surgeon and the surgical team should avoid direct contact with family members and should update them via telephone or video conferencing

The preoperative setting (clinic, office or non-SARS-CoV-2-care [NCC] areas) should consider screening all patients for symptoms of SARS-CoV-2 before their appointment, including temperature checks. All staff and others working in the facility (physicians, nurses, housekeeping, delivery, etc.) should be routinely screen. The American College of Surgeons is clear on this topic.

OrthoEvidence recommends limiting or restricting patient visitors in clinical settings and screening all patients and personnel entering clinics and hospitals.

3. Is there a need for extra disinfecting environmental procedures in between operations?

*Statement* Standard protocols for cleaning and sterilising instruments need to be meticulously applied. Moreover, once the patient has departed from the operating room, this should be left empty for a specific period of time and all high-touch surfaces, including the anaesthetic machine and the anaesthetic work area, should be cleaned and disinfected with an Environmental Protection Agency (EPA) -approved hospital disinfectant. The length of time in between patients depends on the number of air exchanges per hour in the room or space in question. More detailed guidance is available from the US Centers for Disease Control and Prevention (CDC).

*Rationale* The operating room and surrounding exchange areas must be sanitised as soon as possible after each procedure, with particular attention paid to all objects used in caring for patients. Similarly, all areas where SARS-COV-2 patients have transited must be carefully sanitised, too. All personnel must contribute to maintaining a clean environment, including washing floors and surfaces in general. All potentially infected single-use materials should be disposed of in IRHW containers. Reusable materials should be decontaminated, washed, dried and disinfected or sterilised, based on the likelihood of infection. Electromedical equipment (i.e. ventilator, radiological equipment) must be cleaned with a chloro-derivate solution, rinsed and dried, and then disinfected with a chloro-derivate solution in a concentration of ≥ 0.1% or 1000 ppm (parts per million) with contact time superior to 1 min, or as mandated by local guidelines. Full PPE must be worn during the sanitising procedure. Only disposable materials should be used for cleaning (i.e. double gloves, paper towels). Anything disposable kept inside the operating theatre during the procedure must be disposed of in IRHW containers, even if it was not used [7].

According to the American College of Surgeons, facility cleaning policies must be considered in the context of SARS-CoV-2. Cleaning (in all areas) needs to be addressed along the continuum of care (e.g. clinic, preoperative, ORs, workrooms, path-frozen, recovery rooms, wards, ICUs, ventilators, scopes, etc.).

ECDC-Europe recommends regular cleaning followed by disinfection, using hospital disinfectants active against viruses; cleaning in patient rooms is particularly important for frequently touched surfaces. If there is a shortage of hospital disinfectants, decontamination may be performed with 0.1% sodium hypochlorite (dilution 1:50 if household bleach at an initial concentration of 5% is used) after cleaning with a neutral detergent, although no data are available on the effectiveness of this approach against SARS-CoV-2. Surfaces that may become damaged by sodium hypochlorite can be cleaned with a neutral detergent, followed by disinfecting with a 70% ethanol solution.

4. What roles do ultra-clean operating theatres, orthopaedic exhaust suits and ultraviolet light systems play in both the wards/beds and the theatre?

*Statement* Elective orthopaedic theatres and operative procedures, including personal protective equipment, are designed to reduce the risk of surgical site infection. There are no guidelines or protocols published for managing elective hip and knee arthroplasty procedures with respect to laminar flow in the presence of SARS-CoV-2. It is recommended that surgical helmets not be used as primary protection against aerosol and airborne disease. The surgeon should consider wearing an N95 mask as an added precaution when using a surgical helmet in patients considered to be SARS-CoV-2-negative in the preoperative work-up.

Ultraviolet light has no evidence to support its routine use and poses a hazard to operating staff.

*Rationale*

- Health care providers have a duty to protect both patients and staff from cross infection
- The risk of transmission of SARS-CoV-2 between staff and patient is small when both staff and patient have been screened and tested negative. However, transmission would result in having to quarantine staff members if proper PPE has not been used.
- The major transmission risk is aerosol production by power tools.

It is preferable that the operating room have a ventilation system with a minimum of 20 air changes per hour. Filters that are able to remove aerosol particles and droplets can be installed, while there is no actual need for negative-pressure rooms.

Previous studies performed during the SARS epidemic have shown that, commonly used commercially available surgical helmets do not filter particles of 0.02–1 μm in diameter to meet the standard for protective respirators. Unlike powered air-purifying respirators (PAPRs), which draw ambient air through a HEPA filter and blow it over the face, surgical helmets filter air through the hood material itself, placing the surgeon at risk for viral exposure. Modification of the helmet systems using additional 3D printed parts or additional filter materials being taped to the helmets have been reported but have not been tested widely. The manufacturer of one of the most popular helmets cautions against these modifications and warns against increased CO_2_ concentration. N95 masks can be worn under helmets for additional protection. The International Consensus Group (ICM) and the AAHKS Research Committee advises against the use of surgical helmets in the setting of elective procedures.

ESSKA guidelines state that aerosol-generating procedures (AGPs) can be defined as respiratory or surgical. Respiratory AGPs, such as intubation, are a high risk for transmitting respiratory viral infections, such as COVID-19. Surgical AGPs, such as the use of high-speed power tools, are a high risk for transmitting viruses in body fluids and body tissues; SARS-CoV-2 is known to affect all body fluids.

Although there is a debate about the necessity of laminar flow for preventing surgical site infection, there is no doubt that it is beneficial in reducing the risk of transmission of fluids between staff working in the operative field and patients. Although guidelines for surgery, which cover all surgical procedures, do not mention laminar flow and exhaust suits as primary protection against aerosol and airborne disease, their use (including a mask) for elective hip and knee arthroplasty, in which power tools are essential, should be recommended [4, 11, 27, 37].

5. What are the guidelines for personal protective equipment (PPE), in terms of availability and instructions regarding its use and what to wear in relation to the patient’s position in the perioperative chain?

*Statement* During hip and knee arthroplasty, the use of power tools, burrs or electrocautery generates potentially infective aerosol. The major aim should be to avoid transmission of SARS-CoV-2 by aerosolisation of blood or other body fluids. Hence, adequate personal protective equipment should be available and used during surgery. First and foremost, all patients undergoing elective surgery should wear a mask. Surgeons and the entire surgical team who scrub during procedures should ideally wear an exhaust suit, including a mask (preferably N95, filtering face piece [FFP2, or P3] and a face shield). In the absence of a face shield, protective eyewear may be used, but this is a compromise. In addition, scrubs should be frequently changed during the surgical day. Single-use gowns, single-use gloves and hair and shoe covers can also, theoretically, reduce the transmission of the virus [1, 8, 17, 29, 33].

*Rationale* For more in-depth outcomes of the systematic review and expert opinions on this topic, see Hirschmann et al. [16] on PPE use during the SARS-CoV-2 pandemic.

ESSKA Guidelines provide extensive recommendations concerning PPE in “Results”: Recommended Personal Protective Equipment for the Orthopaedic and Trauma Surgeon’.

The American College of Surgeons mentions that PPE guidelines should include PPE recommendations for SARS-CoV-2-positive, PUI and non-SARS-CoV-2 patients for all patient care, including high-risk procedures (e.g. intubation, chest tubes, tracheostomy).

- These should be consistent with CDC and Centers for Medicare and Medicaid (CMS) recommendations for PPEs outside the operating room
- Facilities may consider having all health care workers and staff wear appropriate-level PPE, while patients wear cloth masks during the ramp-up period and possibly beyond

OrthoEvidence recommends that all surgeons working undergo PPE fit testing and review up-to-date sources for training on its appropriate use. Also, contingency plans should be developed for supply chain issues.

The current guidelines of the US CDC, along with US state, county and local regulations/guidelines, specify the following:

- To address asymptomatic and pre-symptomatic transmission, implement source control for everyone entering a health care facility regardless of symptoms:
  - Surgical masks are to be used in all clinical areas by staff
  - Patients may wear either cloth masks or surgical masks
  - For non-clinical areas, patients and staff may wear cloth masks
  - Eye protection worn upon entry to the patient room or care area
- N95 masks or other FDA/NiOSH approved equivalent and eye protection, gowns and gloves should be used for procedures that may lead to aerosolisation of viral particles. N95 with exhaust valves may not provide source control and also should not be used in the operating room.

The BMJ has an overview of the PPE advice on their website, and the revised guidelines from Public Health England recommend that health care workers caring for patients with suspected or confirmed SARS-CoV-2 should “have access to the PPE that protects them for the appropriate setting and context” and that “risk is not uniform and so elements of the updated guideline are intended for interpretation and application dependent on local assessment of risk”.

### Post-operative phase

1. Since the impact on mind and body during joint arthroplasty is significant, do the patients may have lower immunity and be more vulnerable to SARS-CoV-2 infection?

*Statement* As SARS-CoV-2-positive is an absolute contra-indication for elective surgery, the standard enhanced recovery protocol for reducing complications is imperative.

*Rationale* The elderly are at increased risk for adverse events. Frailty, more than age, is related to suboptimal outcomes. In addition to the standard risks, based on the currently available information and clinical expertise, adults 65 years and older, people who are overweight and people with pre-existing medical conditions (such as asthma, diabetes, heart disease) are at higher risk for severe illness and death from SARS-CoV-2.

The patient’s immune function is a major determinant of the disease’s severity, and people with poor immune function, such as older people, are more vulnerable and have higher mortality with SARS-CoV-2 infection [21]. Surgery may not only cause immediate impairment of immune function, but can also induce an early systemic inflammatory response. Similar to the impact of infection with Middle East Respiratory Syndrome Coronavirus (MERS-CoV), the SARS-CoV-2 infected lung can induce and increase the amount of macrophage and neutrophil infiltration, plus increase levels of pro-inflammatory cytokines and chemokines. High levels of circulating inflammatory cytokines have been reported to be correlated with the severity of the illness in patients infected with SARS-CoV-2.

ESSKA Guidelines emphasise that complete planning of the treatment pathway should be made available and carefully discussed with the patient prior to any operation. Care should be taken to only operate on patients when a standardised and adequate post-operative rehabilitation can be assured.

The International Consensus Group (ICM) and the AAHKS Research Committee recommend that the following changes be made in the post-operative care of patients during the pandemic:

- The length of hospital stay for patients should be minimised
- Post-operative rounds by the surgeon may be done using telemedicine, whenever possible
- Patients should be discharged home and transfer to inpatient rehabilitation minimised
- The patient should be instructed on how to perform self-directed physical therapy at home
- Post-discharge visits to the office should be minimised, with the majority of the follow-up being done by telemedicine
- Office visits should be limited to those who are having issues/complications, such as wound healing problems, suspected fracture, stiffness and so on
- Digital health programmes and wearable sensor technologies that allow monitoring of patients will play a larger role in management of patients in the future
- Social distancing should be resumed and at-home visits avoided, unless absolutely essential

OrthoEvidence recommends that patients with substantial co-morbidities and risk factors should be scheduled after healthier patients have been treated and experience has been amassed from the establishment of screening, prevention and treatment protocols.

2. Should a shelter-in-place post-operative period, home care, community nurses or informal care be organised?

*Statement* Ideally, patients should be discharged home with the standard SARS-CoV-2 precautions being taken and only transferred to a nursing home in cases where that is not possible, since higher rates of SARS-CoV-2 may exist in those facilities.

*Rationale* The communal nature of nursing homes and long-term care facilities and the characteristics of the population served (generally older adults, often with underlying medical conditions) put those living in nursing homes at higher risk of infection and severe illness from SARS-CoV-2. Until normality returns, unnecessary human contact should be avoided as much as possible to reduce the potential transmission of SARS-CoV-2 among patients and their family members. All these people, including family members and nurses, can transmit the SARS-CoV-2 virus from one person to another and from one location to another.

The International Consensus Group (ICM) and the AAHKS Research Committee recommend using the general principles of social distancing and minimising contact between individuals. Although no specific studies have been completed relating to the post-operative care of orthopaedic patients, logically one would expect that minimising hospital stays and minimising transfer to rehabilitation facilities or other health care settings are likely to reduce the risk for transmission of the disease.

ESSKA Guidelines highlight that care should be taken to only operate on patients when a standardised protocol for adequate post-operative rehabilitation is in place.

The American College of Surgeons’ advice is to evaluate and discuss the patient’s potential need for a post-acute care facility (rehabilitation medicine, skilled nursing facility, other) before the operation, taking into account the known SARS-CoV-2 outbreaks in the relevant post-acute care facilities.

3. Avoid face-to-face contact for the orthopaedic follow-up and physical therapy guidance
  Consider telemedicine

*Statement* Web-based tools and telemedicine are preferred during this SARS-CoV-2 pandemic and may become the standard in the post-pandemic era.

*Rationale* The use of internet video conferencing in health care settings (telemedicine) is widespread [38], reflecting the normalisation of this mode of communication in society and current health care policy. During this SARS-CoV-2 pandemic, especially, telemedicine is a great tool for minimising physical contact and avoiding SARS-CoV-2 transmission in accordance with general social distancing principles. Telemedicine can be rapidly integrated and easily transitioned to. Similar outcomes to inpatient visits have been reported in rural areas, with good to excellent results in 95% of cases [41]. The combination of web-based platforms and the widespread use of mobile devices allows for reducing post-discharge visits, with early detection and management of post-operative adverse events, including problems related to medication, wound healing and activity. Home exercises for physical therapy can be provided via web-based platforms. Among the limitations of telemedicine is that no physical examinations are possible; no critical assessment of associated conditions and potentially dangerous side effects can be evaluated [39]. This might compromise patient care. All stakeholders using telemedicine must be aware that cybersecurity is also a fundamental issue. The latest reports therefore recommend using secure patient portals instead of popular public services, which can compromise sensitive patient data. Secure patient portals are less vulnerable to cyber-attacks, and patient data can be stored on a secure server. Should popular public services manage to improve their data security guidelines, they might become an option again [41]. Health care payers’, regulatory and legal restrictions and requirements for telemedicine in each country will have to be considered. Each health care facility will have established procedures for this. In the EU and UK, the use of personal devices risks both heavy fines if the law is not meticulously followed and employment problems for people working at a national health care facility.

OrthoEvidence recommends online/telerehabilitation platforms to facilitate remote access while distancing measures are in place.

The International Consensus Group (ICM) and the AAHKS Research Committee recommend that the following changes be made in the post-operative care of patients during the pandemic:

- Post-discharge visits to the office should be minimised, with the majority of the follow-up being done by telemedicine.
- Digital health programmes and wearable sensor technologies that allow monitoring of patients can play a larger role in the management of patients in the future.

The AIOT Joint Statement on COVID-19 Best Practices recommends:

- Minimising imaging requirements
- Avoiding taking images that are unlikely to change patients’ management
- Preventing unnecessary follow-up appointments

Promoting telehealth modalities to increase access for diagnostic and rehabilitation purposes.

- Consider a new approach in wound closure technique in the setting of the SARS-CoV-2 pandemic

*Statement* Post-operative in-office visits should be minimised, and digital health programmes allowing health care providers to closely monitor patients remotely should be supported. Self-administered wound management should become the standard of care for patients in the post-pandemic era.

*Rationale* The goal of this approach is to avoid a follow-up visit at 2 weeks from the original operation. Modern, fast-track wound closure techniques need to be applied in the wake of SARS-CoV-2. Capsular closure has traditionally been performed using interrupted knotted sutures; in recent years, the number 1 knotless barbed suture has been introduced, allowing for a running technique aimed at reducing closure time, while still providing excellent results. The subcutaneous fat layer, when substantial, should be closed using synthetic, absorbable, braided sutures or bidirectional barbed sutures. Barbed sutures also demonstrate significantly less bacterial adherence than conventional braided sutures. Skin closure should be performed using 2-octyl cyanoacrylate (glue) and polyester mesh with or without monocryl running suture. This technique allows for patient self-management of the wound and offers superior cosmetic outcomes to staples. The use of video-based digital technologies will allow the health care provider to monitor the wound healing process remotely [20, 23, 36].

4. What is the advice for patients who develop SARS-CoV-2 symptoms?

*Statement* In the event a patient develops SARS-CoV-2 symptoms or comes into contact with a SARS-CoV-2-positive person, they should seek health care advice. The patient should use the system in place in their country to seek the relevant advice. This may be through their primary care physician or an online or telephone advice service. Their orthopaedic surgeon and hospital should also be notified.

*Rationale* It is important to recognise the danger signs. If early symptoms of SARS-CoV-2 are missed, patients, particularly the elderly, may deteriorate rapidly before getting needed care. If the patient has a fever or acute respiratory illness, they should immediately seek health advice.

OrthoEvidence recommends online or telerehabilitation platforms; this will facilitate early detection of SARS-CoV-2 symptoms by the surgeon or hospital.

The WHO advice is:

- If you have minor symptoms, such as a slight cough or a mild fever, there is generally no need to seek medical care. Stay at home, self-isolate and monitor your symptoms. Follow national guidance on self-isolation
- However, if you live in an area with malaria or dengue fever, it is important that you do not ignore symptoms of fever. Seek medical help. When you attend the health facility, wear a mask if possible, keep at least 1-m distance from other people and do not touch surfaces with your hands. If it is a child who is sick, help the child stick to this advice
- Seek immediate medical care if you have difficulty breathing or pain or pressure in the chest. If possible, call your health care provider in advance, so they can direct you to the right health care facility.

The International Consensus Group (ICM) and the AAHKS Research Committee recommend that if a patient is positive for SARS-CoV-2, as determined by an RT-PCR test after a surgical procedure, all health care workers who came into contact with the patient without using PPE, and are not known to have antibodies against SARS-CoV-2, should be tested and quarantined until their test results become available. Decisions regarding the need to quarantine staff should be made in tandem with the hospital infection control team and employee health department. The patient should also be isolated and any contact with the patient should be with the use of full PPE.

The American College of Surgeons mentions guidelines and considerations for post-operative SARS-CoV-2 testing of symptomatic patients or patients under investigation (PUI). Atelectasis, fevers, et cetera, are not uncommon in the post-operative course. Establishing operational guidelines for SARS-CoV-2 testing in these patients and concurrent testing results should be considered.

5. Are special adjustments needed for post-op SARS-CoV-2 prophylaxis?

*Statement* Since SARS-CoV-2-positive is an absolute contra-indication for elective surgery, the standard enhanced recovery protocol and SARS-CoV-2 preventive measures are imperative. Time allowing, all surgical patients should apply social distancing principles for the first 2 weeks after the operation and self-quarantine in their home.

*Rationale* To date, there is no known SARS-CoV-2 prophylaxis advice; the standard enhanced recovery protocols and SARS-CoV-2 preventive actions should prevail.

To prevent infection and slow transmission of SARS-CoV-2, do the following:

- Wash your hands regularly with soap and water or clean them with alcohol-based hand rub
- Maintain at least a 1.5-m distance between you and people coughing or sneezing
- Avoid touching your face
- Cover your mouth and nose when coughing or sneezing
- Stay home if you feel unwell
- Refrain from smoking and other activities that weaken the lungs
- Practice physical distancing by avoiding unnecessary travel and staying away from large groups of people.

The International Consensus Group (ICM) and the AAHKS Research Committee do not recommend that antibiotic or venous thromboembolism (VTE) prophylaxis be altered in patients undergoing elective surgery during the SARS-CoV-2 pandemic.

## Discussion

The recommendations have been based on the available evidence and have been validated by a survey of the EHS and EKA membership [19]. They have been set out to follow the sequence of events that take a patient to an operating theatre for an elective primary hip or knee arthroplasty, and then into the recovery period. Implementation of the recommendations will require a multidisciplinary approach that includes hospital management, and includes the production of guidelines, staff training, patient information material, and finance. However the goal is to undertake operations effectively and safely for the patients and the staff. At the same time planning needs to begin on how to manage a new phase of SARS-CoV-2.

## Conclusions

The recommendations can be summarised as:

1. National guidance for the management of the SARS-CoV-2 pandemic should be adhered to.
2. National and local guidance for the pathway for elective arthroplasty should be followed.

In addition:

(i) Patient information materials should now include information on what the patient should do if they develop SARS-CoV-2 symptoms or become SARS-CoV-2 positive whilst on the elective arthroplasty pathway.
(ii) Patients should only be accompanied by their primary caregiver during their elective hip or knee arthroplasty pathway.
(iii) The appropriate facilities, workforce, testing and supplies for all the equipment needed must be in place.
(iv) Agreed protocols should be in place for selecting suitable patients, taking into consideration the resources available to cater to their needs on an individual basis.
(v) A robust testing regime for SARS-CoV-2 for both patients and staff should be in place and incorporated into the protocols for the patient journey. Being SARS-CoV-2-positive is an absolute contra-indication for primary elective hip and knee arthroplasty.
(vi) Enhanced recovery protocols are encouraged, with a further reduction in the inpatient stay implemented.
(vii) To reduce the risk of aerosolisation to staff, the use of spinal anaesthetic techniques is encouraged and should be included in the patient information material. An agreed policy must be in place on how to manage patients who can only undergo a general anaesthetic, which may include this being a contra-indication for surgery.
(viii) Theatre staff should use personal protective equipment (PPE) in line with national and local policies. Laminar flow theatres are encouraged, and, if available, exhaust suits with a mask should be used for the operating team.
(ix) Primary elective hip and knee arthroplasty patients should be physically isolated from patients admitted with trauma or for other emergencies, including the sick elderly.
(x) Ideally, patients should be discharged directly back to their normal residence. Patients should be advised to maintain social distancing for 2 weeks following discharge.
(xi) Post-operative rehabilitation should be implemented during the inpatient stay, with remote follow-up as the norm after discharge.
(xii) Follow-up visits should be minimised and remote follow-up instituted with the relevant approvals with respect to Patient Confidentiality and Data Protection in place.

## References

[1] Aggarwal N (2020). Importance of social distancing: modeling the spread of 2019-nCoV using susceptible-infected-quarantined-recovered-t model. MedRxiv.

[2] Al-Muharraqi MA (2020). Testing recommendation for COVID-19 (SARS-CoV-2) in patients planned for surgery—continuing the service and 'suppressing' the pandemic [published online ahead of print, 2020 Apr 13]. Br J Oral Maxillofac Surg.

[3] Aminian A, Kermansaravi M, Azizi S, Peyman A, Safamanesh S, Mousavimaleki A (2020). Bariatric surgical practice during the initial phase of COVID-10 outbreak. Obes Surg.

[4] Anon (2020) Covid-19: Good Practice for Surgeons and Surgical Teams. Royal College of Surgeons of England 2020. https://www.rcseng.ac.uk/standards-and-research/gsp/. Accessed 7 May 2020

[5] Brown TS, Bdard NA, Rojas EO, Prieto H, Parvizi J (2020). The effect of the COVID-19 pandemic on electively scheduled hip and knee arthroplasty patients in the United States. J Arthroplasty.

[6] Centers for Medicare & Medicaid Services (CMS) Recommendations Re-opening Facilities to Provide Non-emergent Non-COVID-19 Healthcare: Phase. https://www.cms.gov/files/document/covid-flexibility-reopen-essential-non-covid-services.pdf. Accessed 5 May 2020

[7] Coccolini F, Perrone G, Chiarugi M, Di Marzo F, Ansaloni L, Scandroglio I (2020). Surgery in COVID-19 patients: operational directives. World J Emerg Surg.

[8] Coimbra R, Edwards S, Kurihara H, Bass GA, Balogh ZJ, Tilsed J (2020). European Society of Trauma and Emergency Surgery (ESTES) recommendations for trauma and emergency surgery preparation during times of COVID-19 infection. Eur J Trauma Emerg Surg.

[9] D’Apolito R, Faraldi M, Ottaiano I, Zagra L (2020). Disruption of arthroplasty practice in an orthopaedic Center in Northern Italy during COVID-19 pandemic. J Arthroplasty.

[10] ESSKA (2020) Which patients should you begin with for elective orthopaedic surgery? https://cdn.ymaws.com/www.esska.org/resource/resmgr/covid-19/Surgical_priorities.pdf. Accessed 1 May 2020

[11] Evans RP (2011). Current concepts for clean air and total joint arthroplasty: laminar airflow and ultraviolet radiation. Clin Orthop Relat Res.

[12] Fineberg HV (2020). Ten weeks to crush the curve. N Engl J Med.

[13] Fujihara N, Lark ME, Fujihara Y, Chung KC (2017). The effect of economic downturn on the volume of surgical procedures: a systematic review. Int J Surg.

[14] Ghomrawi HMK, Mushlin AI, Kang R, Banerjee S, Singh JA, Sharma L (2020). Examining timeliness of total knee arthroplasty among patients with knee osteoarthritis in the US: results from the OAI and MOST longitudinal cohorts. J Bone Jt Surg Am.

[15] Guan W-J, Liang W-H, Zhao Y, Liand H-R, Chen Z-S, Li Y-M (2020). Comorbidity and its impact on 1590 patients with Covid-19 in China: a Nationwide analysis. Eur Respir J.

[16] Hirschmann MT, Hart A, Henckel J, Sadoghi P, Seil R, Mouton C (2020). COVID-19 coronavirus: recommended personal protective equipment for the orthopaedic and trauma surgeon. Knee Surg Sports Traumatol Arthrosc.

[17] Klompas M, Morris CA, Sinclair J, Pearson M, Shenoy ES (2020). Universal masking in Hospitals in the Covid-19 era. N Engl J Med.

[18] Koenig L, Zhang Q, Austin MS, Demiralp B, Fehring TK, Feng C (2016). Estimating the societal benefits of THA after accounting for work status and productivity: a Markov model approach. Clin Orthop Relat Res.

[19] Kort NP, Gómez Barrena E, Bédard M, Donell S, Epinette J-A, Gomberg B (2020). A survey of recommendations for resuming elective hip and knee arthroplasty after the first phase of the SARS-CoV-2 pandemic: The European Hip Society and European Knee Associates. Knee Surg Sports Traumatol Arthrosc.

[20] Krebs VE, Elmallah RK, Khlopas A, Chughtai M, Bonutti P, Roche M (2018). Wound closure techniques for total knee arthroplasty: an evidence-based review of the literature. J Arthroplasty.

[21] Lei S, Jiang F, Su W, Chen C, Chen J, Mei W (2020). Clinical characteristics and outcomes of patients undergoing surgeries during the incubation period of COVID-19 infection. E Clin Med.

[22] Li Y, Fu J-B, Li K-F, Liu J-N, Wang H-L, Liu L-J (2020). Transmission of COVID-19 in the terminal stages of the incubation period: a familial cluster. Int J Infect Dis.

[23] Lie SA, Wong SW, Wong LT, Wong TGL, Chong SY (2020). Practical considerations for performing regional anesthesia: lessons learned from the COVID-19 pandemic. Can J Anesth.

[24] Meneghini RM (2020). Resource reallocation during the COVID-19 pandemic in a suburban hospital system: implications for outpatient hip and knee arthroplasty. J Arthroplasty.

[25] Mouton C, Hirschmann M, Ollivier M, Seil R, Menetrey J (2020) COVID-19—ESSKA Guidelines and Recommendations for Resuming Elective Surgery. https://cdn.ymaws.com/www.esska.org/resource/resmgr/covid-19/COVID-guidelines-Q&A.pdf. Accessed 1 May 2020 10.1186/s40634-020-00248-4 PMC7220621 32405872

[26] Mujica-Mota RE, Watson LK, Tarricone R, Jager M (2017). Cost-effectiveness of timely versus delayed primary total hip arthroplasty in Germany: a social health insurance perspective. Orthop Rev.

[27] National Institute for Health and Care Excellence (2020) Joint arthroplasty (primary): hip, knee and shoulder. NICE guideline [NG157] www.nice.org.uk/guidance/ng157. Accessed 7 May 2020

[28] Park SY, Kim YM, Yi S, Lee S, Na BJ, Kim CB (2020). Coronavirus disease outbreak in call center, South Korea. Emerg Infect Dis.

[29] Parvizi J, Gherke T, Krueger JA, Chisari E, Citak M (2021). Resuming elective surgery during the COVID-19 pandemic. J Bone Jt Surg Am.

[30] Patel R, Babady E, Theel ES, Storch GA, Pinsky BA, St George K (2020). Report from the American Society for Microbiology COVID-19 International Summit, 23 March 2020: value of diagnostic testing for SARS–CoV-2/COVID-19. mBio.

[31] Pollock M, Somerville L, Firth A, Lanting B (2016). Outpatient total hip arthroplasty, total knee arthroplasty, and unicompartmental knee arthroplasty: a systematic review of the literature. JBJS Rev.

[32] Prachand VN, Milner R, Angelos P, Posner MC (2020). Medically necessary, time-sensitive procedures: scoring system to ethically and efficiently manage resource scarcity and provider risk during the COVID-19 pandemic. J Am Coll Surg.

[33] Rodrigues-Pinto R, Sousa R, Oliveira A (2020). Preparing to perform trauma and orthopaedic surgery on patients with COVID-19. J Bone Jt Surg Am.

[34] Rossi MD, Eberle T, Roche M, Waggoner M, Blake R, Burwell B, Baxter A (2009). Delaying knee arthroplasty and implications on early postoperative outcomes: a pilot study. Orthopedics.

[35] Ruiz D, Koenig L, Dall TM, Gallo P, Narzikul A, Parvizi J (2013). The direct and indirect costs to society of treatment for end-stage knee osteoarthritis. J Bone Jt Surg Am.

[36] Sanger PC, Simianu VV, Gaskill CE, Armstrong CAL, Hartzler AL, Lordon RJ (2017). Diagnosing surgical site infection using wound photography: a scenario-based study. J Am Coll Surg.

[37] Smit S (2010). Guidelines for surgery in the HIV patient. CME.

[38] Smith WR, Atala AJ, Terlecki RP, Kelly EE, Matthews CA (2020). Implementation guide for rapid integration of an outpatient telemedicine program during the COVID-19 pandemic. Acad Oncol Nurse Patient Navig.

[39] Sundaram K, Piuzzi NS, Patterson BM, Stearns KL, Krebs VE, Mont MA (2020). Skin closure with 2-octyl cyanoacrylate and polyester mesh after primary total knee arthroplasty offers superior cosmetic outcomes and patient satisfaction compared to staples: a prospective trial. Eur J Orthop Surg Traumatol.

[40] Soffin EM, YaDeau JT (2016). Enhanced recovery after surgery for primary hip and knee arthroplasty: a review of the evidence. Br J Anaesth.

[41] Tanaka MJ, Oh LS, Martin SD, Berkson EM (2020). Telemedicine in the era of COVID-19: the virtual orthopaedic examination. J Bone Jt Surg Am.

[42] Wainwright TW, Gill M, McDonald DA, Middleton RG, Reed M, Sahota O (2020). Consensus statement for perioperative care in total hip arthroplasty and total knee arthroplasty surgery: Enhanced Recovery After Surgery (ERAS(®))Society recommendations. Acta Orthop.

[43] White House (2020) Guideline Opening Up America Again. https://www.whitehouse.gov/openingamerica/#criteria. Accessed 15 May 2020

[44] Zhu S, Qian W, Jiang C, Ye C, Chen X (2017). Enhanced recovery after surgery for hip and knee arthroplasty: a systematic review and meta-analysis. Postgrad Med J.

